# A pilot study of the S-MAP (Solutions for Medications Adherence Problems) intervention for older adults prescribed polypharmacy in primary care: study protocol

**DOI:** 10.1186/s40814-019-0506-6

**Published:** 2019-10-22

**Authors:** D. E. Patton, J. J. Francis, E. Clark, F. Smith, C. A. Cadogan, C. Ryan, C. M. Hughes

**Affiliations:** 1 School of Pharmacy, Queen’s University Belfast, Belfast, Northern Ireland; 2 School of Health Sciences, City University of London, London, UK; 3 School of Pharmacy, University College London, London, UK; 4 School of Pharmacy, Royal College of Surgeons in Ireland, Dublin, Ireland; 5 School of Pharmacy & Pharmaceutical Sciences, Trinity College Dublin, Dublin, Ireland

**Keywords:** Medication adherence, Polypharmacy, Theory, Behaviour change, Community pharmacists, Complex intervention, Pilot study, Process evaluation, Technology

## Abstract

**Background:**

Adhering to multiple medications as prescribed is challenging for older patients (aged ≥ 65 years) and a difficult behaviour to improve. Previous interventions designed to address this have been largely complex in nature but have shown limited effectiveness and have rarely used theory in their design. It has been recognised that theory (‘a systematic way of understanding events or situations’) can guide intervention development and help researchers better understand how complex adherence interventions work. This pilot study aims to test a novel community pharmacy-based intervention that has been systematically developed using the Theoretical Domains Framework (12-domain version) of behaviour change.

**Methods:**

As part of a non-randomised pilot study, pharmacists in 12 community pharmacies across Northern Ireland (*n* = 6) and London, England (*n* = 6), will be trained to deliver the intervention to older patients who are prescribed ≥ 4 regular medicines and are non-adherent (self-reported). Ten patients will be recruited per pharmacy (*n* = 120) and offered up to four tailored one-to-one sessions, in the pharmacy or via telephone depending on their adherence, over a 3–4-month period. Guided by an electronic application (app) on iPads, the intervention content will be tailored to each patient’s underlying reasons for non-adherence and mapped to the most appropriate solutions using established behaviour change techniques. This study will assess the feasibility of collecting data on the primary outcome of medication adherence (self-report and dispensing data) and secondary outcomes (health-related quality of life and unplanned hospitalisations). An embedded process evaluation will assess training fidelity for pharmacy staff, intervention fidelity, acceptability to patients and pharmacists and the intervention’s mechanism of action. Process evaluation data will include audio-recordings of training workshops, intervention sessions, feedback interviews and patient surveys. Analysis will be largely descriptive.

**Discussion:**

Using pre-defined progression criteria, the findings from this pilot study will guide the decision whether to proceed to a cluster randomised controlled trial to test the effectiveness of the S-MAP intervention in comparison to usual care in community pharmacies. The study will also explore how the intervention components may work to bring about change in older patients’ adherence behaviour and guide further refinement of the intervention and study procedures.

**Trial registration:**

This study is registered at ISRCTN: 10.1186/ISRCTN73831533

## Background

Polypharmacy, often defined as the use of multiple medicines [1], is increasingly accepted as the new paradigm for prescribing in older adults (≥ 65 years) [2]. However, this can give rise to challenges with adherence, a behaviour that is influenced by multiple factors, and has proved resistant to interventions [3]. Increasing healthcare costs and wastage of medications resulting from non-adherence have major financial implications for healthcare systems. A 2012 report from the Institute for Healthcare Informatics estimated that total avoidable costs globally from non-adherence across 186 countries including the United Kingdom (UK) were approximately US$270 billion per year [4]. In addition, for patients with long-term conditions, non-adherence may result in treatment failures, poor disease control and reduced quality of life [5]. To improve adherence and health outcomes, a complex intervention with multiple interacting components is warranted. However, previous complex interventions have shown only limited effectiveness [3]. Furthermore, the intervention components are rarely reported in sufficient detail to enable others to replicate the studies or implement the interventions into practice. To maximise effectiveness, the UK Medical Research Council (MRC) recommends that complex interventions are designed, evaluated and reported in a systematic and rigorous way [6].

There are three key gaps in adherence research to date. First, it is generally unclear how intervention components have been selected for inclusion in complex adherence interventions and, without a theoretical underpinning, it is difficult to understand their mechanisms of action [7]. This has been highlighted in a systematic review of adherence interventions delivered to older patients prescribed polypharmacy, which reported that only a limited number of studies used theory to guide intervention development [7]. A theory has previously been defined by Glanz and Rimer [8] as ‘(…) a systematic way of understanding events or situations. It is a set of concepts, definitions, and propositions that explain or predict these events or situations by illustrating the relationship between variables.’ Theories can facilitate researchers’ understanding of health behaviours by firstly explaining and predicting behaviour (how, when and why it occurs) and secondly, helping researchers identify key influences on behaviour that can be targeted for behaviour change. Secondly, interventions are rarely tailored to each patient’s needs, despite findings that adherence problems are specific to individual patients [9, 10]. This has been illustrated in a meta-analysis conducted by Conn and Ruppar [11] which found that only 9 out of 771 adherence interventions delivered to adult patients (≥ 18 years) were tailored on an individual basis. In addition, technology has been increasingly employed in the field of adherence, for example, through the use of electronic reminders, [12] although its role in facilitating individual-level tailoring of complex adherence interventions requires further investigation.

To address the evidence gaps noted above, a novel theory-based tailored intervention has been systematically developed in line with the MRC’s complex intervention framework [6, 10]. The Theoretical Domains Framework (TDF) of behaviour change (12-domain version) [13] was used in a qualitative focus group study to gain an in-depth understanding of older patients’ adherence behaviour [10]. This work led to the identification of eight key behavioural determinants (theoretical domains) to target to improve older patients’ adherence (e.g. ‘motivation and goals’, ‘behavioural regulation’, ‘social influences’) and 11 behaviour change techniques (BCTs) (e.g. ‘goal-setting’, ‘self-monitoring’, ‘social support-unspecified’) that could be the ‘active ingredients’ of the intervention [14, 15]. These 11 BCTs were combined into an intervention which was subsequently tested in a small-scale feasibility study involving two community pharmacies in Northern Ireland (NI) with five patients recruited per pharmacy (previously known as the IDentification of Medication Adherence Problems (ID-MAP) intervention; 10.1186/ISRCTN17966504, [16]). Community pharmacists were selected to deliver this intervention due to their accessibility to patients in the primary care setting and frequency of contact with patients [10]. The intervention was guided by a paper-based adherence assessment tool that was designed to assist pharmacists in identifying adherence problems and selecting tailored solutions (i.e. BCTs) to deliver to patients. Although this feasibility study demonstrated the usability and acceptability of the intervention from the viewpoint of older patients and pharmacists, it highlighted the need for modifications to the intervention, such as an electronic assessment tool to guide intervention tailoring, and to study procedures. Consequently, the study described in the current protocol aims to test a refined version of the intervention—Solutions for Medications Adherence Problems (S-MAP) intervention—and modified study procedures in a larger sample of community pharmacies in two geographical areas (NI and London, England) as part of a non-randomised pilot study. The objectives of the study are to

1. Test the feasibility of methods for recruiting and retaining community pharmacies and patients
2. Develop a web-application (app) to support intervention delivery including an electronic version of the adherence assessment tool to guide intervention tailoring
3. Design and deliver a training programme for pharmacy staff
4. Deliver the S-MAP intervention in the community pharmacy setting in NI and London
5. Undertake a process evaluation (exploring fidelity, acceptability and mechanisms of action)
6. Explore the appropriateness and suitability of selected outcome measures

This pilot study will provide evidence to guide the decision on whether to proceed to a definitive cluster RCT (cRCT) to explore effectiveness of the S-MAP intervention in comparison to usual care in community pharmacies.

## Methods/design

### Study design

This study is a multi-centre non-randomised pilot study consisting of an intervention group only (i.e. no control group). Ethical approval was granted by the Office of Research Ethics Committees for Northern Ireland (REC reference: 17/NI/0193) and the Health Research Authority (IRAS ID: 234121). The information reported in this protocol follows recommendations from the SPIRIT (Standard Protocol Items: Recommendations for Interventional Trials) 2013 statement [17]. A completed SPIRIT checklist can be found in Additional file 1.

### Pharmacy sampling and recruitment

The study will be carried out in 12 community pharmacies in the UK. Maximum variation sampling, a form of purposive sampling that aims to identify a diverse range of participants, will be employed. Six pharmacies will be selected from the five Health and Social Care Trusts (HSCT) areas in NI and six pharmacies selected from six (out of 32) Clinical Commissioning Groups (CCGs) in London. To ensure variation in the sample, the six CCGs will be selected from areas of both low and high levels of social deprivation in London. A single UK-wide measure of social deprivation is not currently readily available [18]. Therefore, the Index of Multiple Deprivation (where a rank of 1 indicates the most deprived area and 9 indicates the least deprived area) will be employed to assess social deprivation levels of areas served by the community pharmacies in London. The NI Multiple Deprivation Measure (where 1 indicates the most deprived area and 890 indicates the least deprived area) will be employed for NI-based pharmacies. As this data will solely be used for reporting the level of variation in the sample (and not for data analysis purposes) no adjustment techniques will be used.

This study also aims to achieve variation in the sample by selecting pharmacies in both rural and urban areas in NI (data source: [19]) and including pharmacies that are independently owned or part of small or large chains. The community pharmacy landscapes differ somewhat in NI and England (i.e. there are very few chains comprising > 100 pharmacies in NI [20], whereas this is more common in England [21]). For that reason, region-specific definitions will be used for categorising pharmacies as being independently owned or part of small or large chains (see Table 1).

**Table 1:** Definitions for pharmacy types in Northern Ireland (NI) and London, England

Type of pharmacy	Definition for NI [20]	Definition for London [21]
Independently owned	1–3 pharmacies	1–5 pharmacies
Small chain	4–9 pharmacies	6–99 pharmacies
Large chain	≥ 10 pharmacies	≥ 100 pharmacies

Pharmacies will be contacted initially via a letter seeking expressions of interest (via a reply slip). A researcher will contact those expressing interest to provide further details. If insufficient reply slips are returned, the researcher will telephone pharmacies to try and recruit the required number. If required, the research team will use personal contacts, local networks and social media to advertise the study and enhance recruitment.

In order to meet the inclusion criteria, community pharmacies must have a private consultation area, Wi-Fi/printing facilities and be registered with the appropriate professional body (e.g. General Pharmaceutical Council). Interested pharmacists will be provided with an information sheet and consent form and a researcher will arrange a meeting to discuss participation. Multiple pharmacists in a single pharmacy can participate provided they each give informed consent. All pharmacists must be employed within the pharmacy on a regular basis (i.e. not a locum pharmacist) and undertake training in study procedures and intervention delivery (see the ‘Training package overview’ section below). Participating pharmacists will be provided with a certificate of participation for continuing professional development purposes and each pharmacy will be offered an honorarium for participation [£500 (NI)/£600 (London)]. As a further incentive, pharmacies will receive an additional £30 for each patient to whom they deliver the intervention (up to a maximum of £300). Where possible, pharmacists will delegate pre-specified tasks (e.g. recruitment of patients) to pharmacy support staff, although pharmacists will be responsible for delivering the intervention to patients. In order to participate in the study, support staff must have completed, as a minimum, an accredited Medicines Counter Assistant course (or equivalent). They will be given a study information sheet and asked to provide written informed consent and undertake training in study procedures (see the ‘Training package overview’ section).

### Patient screening and recruitment

There will be two stages to screening patients for participation in the study. In stage 1, patient medication records (PMR) will be used to identify patients aged 65 years or older who are prescribed ≥ 4 regular medicines (excluding ‘when required’ dosing or variable dosing medications e.g. take one or two tablets daily) and live in their own home (i.e. not a care home). Twelve months of dispensing data must be available on the pharmacy’s PMR system for the patient to be eligible (for outcome assessment purposes, see the ‘Outcome data collection’ section). Patients who are prescribed medications for the treatment/management of dementia (e.g. donepezil) or who are unable to provide informed consent will be excluded as the intervention has not been designed to account for the additional challenges these patients are likely to face.

Following this initial screen, in stage 2, patients will be approached in the pharmacy by trained pharmacy staff or mailed letters inviting them to complete an adherence screening questionnaire (and provide consent for this activity). The screening questionnaire consists of two self-reported measures of adherence [validated Medication Adherence Reporting Scale-5 item (MARS-5) and one item from the Lu et al. instrument] [22, 23]. This aims to identify patients who have adherence difficulties, as research has shown that interventions are more effective when targeted at non-adherent patients [11]. Only patients who are identified as non-adherent via the self-report measure will be invited to take part in the study (questionnaire scoring information available from authors upon request). Pharmacy staff will also confirm that the patient only attends their pharmacy for regular medications. Patients who attend multiple pharmacies will be excluded as complete dispensing records are required for outcome assessment. Eligible patients will be invited to attend their first session of the intervention in the pharmacy (see the ‘Intervention delivery’ section) at which point the pharmacist will ensure informed consent has been given.

This pilot study does not aim to assess effectiveness. Therefore, a sample size calculation has not been undertaken. Each pharmacy will be asked to recruit ten older patients who meet the eligibility criteria listed above as this has been deemed sufficient (based on the extensive experience of the research team and experience in other similar studies) to meet the objectives of this pilot study as described above (120 patients in total). To enhance recruitment, the study will be advertised to patients via flyers/posters displayed in the pharmacy, or on their social media platforms. This study aims to pilot different strategies, such as approaching patients in the pharmacy or via letter, to identify the most feasible strategy for patient screening and recruitment. At the time of submission of this paper, patient recruitment had commenced and an amendment to the study screening processes described above had been implemented. Details of the amended procedures are provided later under the ‘Study amendments’ section.

### Intervention specification

As referred to above, previous qualitative work undertaken by the research team [10] identified 11 BCTs (see Table 2) that were selected to be the proposed ‘active ingredients’ of the patient-targeted intervention and tested in a small-scale feasibility study [16]. Following this, a BCT validation exercise was conducted to ensure an accurate specification of the intervention (unpublished work). This involved coding descriptions of the intervention content using the BCT Taxonomy version 1 [24], by four members of the research team (SC, CC, JF, EC) who were not directly involved in the original mapping from TDF domains to BCTs. Four additional BCTs were identified in this exercise (see Table 2) and have been added to the intervention specification, bringing the total number of BCTs in the S-MAP intervention to 15.

**Table 2:** Behaviour change techniques (BCTs) that will be delivered to older patients as part of the S-MAP intervention

Behaviour change technique (BCT)	Specification for BCT delivery as part of the S-MAP intervention	Context in which the BCT be delivered? (‘core’ or ‘optional’ BCT)^a^
Problem-solving^b^	The pharmacist will prompt the patient to think of factors that influence their medication-taking behaviour (e.g. being away from home) and encourage the patient to select solutions to overcome any barriers or act as facilitators of the behaviour.	All non-adherent patients (core)^a^
Self-monitoring	Patients will be asked to monitor their medication use on a daily basis using a medication diary. This will include a list of the patient’s prescribed medications.	
Feedback on behaviour	Based on a review of the patient’s medication diary, the pharmacist will provide feedback on the patient’s individual adherence at follow-up sessions. For example, the pharmacist might say, ‘You managed to take all of your medicines on weekdays but missed some at weekends’.	
Social support (unspecified)	The pharmacist will provide, or identify others (e.g. family) who can provide, general encouragement to patients with regards to taking their medications as prescribed.	
Social reward^b^	The pharmacist will praise patients who have improved adherence and encourage continued adherence.	Patients in whom adherence has improved (optional)^a^
Goal-setting (behaviour)	The pharmacist will assist patients in setting and writing down an adherence-related goal that specifies a behaviour that will be done. For example, ‘I will use my preventer inhaler every day’.	Patients deemed non-adherent at follow-up sessions (optional)^a^
Action planning	A personalised plan to achieve the goal(s) set will be developed collaboratively by the patient and pharmacist. This plan can include the time, place or how often the behaviour is performed. For example, ‘When it is 9 pm and I am brushing my teeth, then I will take my simvastatin’.	
Review of behaviour goal	The pharmacist and patient together will review the adherence-related goal set at the previous session and re-set or modify this. For example, if the previous goal (‘I will use my preventer inhaler every day’) was too ambitious, then the goal could be modified to one that is more achievable (‘I will use my preventer inhaler at least six days each week’).	
Social support (practical)^b^	Where necessary, additional practical support from family/friends or other healthcare professionals will be arranged. For example, family members could help the patient with organising medications into a weekly pill reminder box.	Tailored based on adherence assessment and patient need (optional)^a^
Goal setting (outcome)	The pharmacist will assist patients who have low motivation in setting and writing down a goal that focuses on the positive outcomes of adherence. For example, ‘My goal is to stay out of hospital’ or ‘My goal is to have more pain free days’.	
Review of outcome goal	The pharmacist and patient together will review the outcome goal that was set at the previous session. The goal will be re-set or modified. For example, it might be that the original outcome goal is not achieved by better adherence but the patient notices another unexpected benefit and decides to focus on that instead.	
Information about health consequences	The pharmacist will inform patients about the benefits of taking their medications as prescribed and the risks associated with non-adherence. Patient leaflets have been designed as part of the intervention to facilitate discussions around medication concerns and generic medications and will be given to patients as part of the intervention if deemed appropriate.	
Prompts and cues	The pharmacist will ask about the contexts in which forgetting is more likely and make suggestions about possible prompts. For example, a patient who routinely forgets their bedtime medications could benefit from linking medication taking to brushing their teeth.	
Restructuring the physical environment	Patients may be advised to change where they store their medications or alter their home environment to facilitate adherence. For example, patients may be advised to store their night-time medications in their bedroom to facilitate adherence.	
Adding objects to the environment^b^	For patients who have difficulties with any aspect of the medication packaging or formulation/regimen and would like support, the pharmacist will provide more appropriate packaging or recommend changes to the prescriber. For example, if the patient has difficulty opening child-resistant bottle caps, then the pharmacist could supply non-child resistant bottle caps.	

^a^‘Core’ BCTs are recommended for delivery to all non-adherent patients in the study. ‘Optional’ BCTs are delivered based on each individual patient’s needs including the underlying reasons for non-adherence and improvements in adherence scores

^b^New BCT labels identified from the validation coding exercise (unpublished work)

### Web-application (app) development overview

In the previous feasibility study, all intervention and study materials were paper-based which was time-consuming, burdensome and sometimes impractical in a busy healthcare environment [16]. To overcome this in the current pilot study, pharmacies will be provided with iPads to assist with intervention delivery. A web-application (app) will be developed (accessible on the iPad) that will act as a decision support tool during the intervention. The app will support pharmacists in identifying adherence problems and mapping these to potential adherence solutions (i.e. BCTs). Doing so will assist the tailoring of the intervention content to each individual patient’s needs. Pharmacists will be trained to use the app as part of the above-mentioned training package. For practical reasons, paper-based forms will be used for study procedures (e.g. study information sheets, questionnaires).

### Intervention delivery

Research has shown that older patients’ reasons for non-adherence are often individual and, therefore, it would be inappropriate, or unnecessary, to deliver all 15 BCTs to each patient [10]. Consequently, the S-MAP intervention is intended to be a ‘personalised’ intervention whereby the intervention is tailored to the individual needs of each patient. Some BCTs, such as ‘problem-solving’, ‘self-monitoring’ and ‘feedback on behaviour’, are potentially relevant to all patients, irrespective of why they are non-adherent. For example, a brief self-monitoring period using a medication diary could act to help establish new routines (for patients who are unintentionally non-adherent), reinforce the importance of adherence or act as a platform for pharmacists to engage patients (who are intentionally non-adherent) in discussions about the underlying reasons for non-adherence. These BCTs have been termed ‘core’ BCTs [16]. Some BCTs are unlikely to benefit all patients, for example, a patient who intentionally decides to stop taking their medication because they are worried about potential side effects is unlikely to benefit from the BCT ‘prompts and cues’ which addresses the problem of forgetting. Alternatively, a patient who is already aware of the health consequences of non-adherence, but simply forgets to take medications, is unlikely to benefit from the BCT ‘health consequences’. These types of BCTs will, therefore, be recommended for delivery based on an assessment of patients’ underlying reasons for non-adherence and reported adherence levels which will be guided by the app. For example, the BCT ‘social reward’ (i.e. verbal praise) will only be delivered to those patients in whom there has been an improvement in adherence. These have been termed ‘optional’ BCTs [16]. Table 2 indicates the context in which each BCT will be delivered as part of the intervention and whether it has been deemed a ‘core’ or ‘optional’ BCT.

**Fig. 1:**
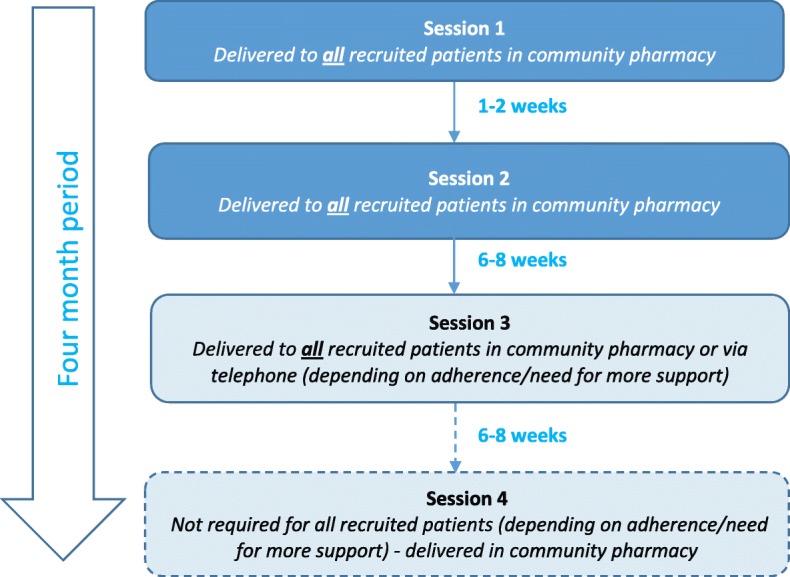
Overview of the session flow in the S-MAP intervention

Adherence problems for patients prescribed multiple medications to treat a variety of conditions can range in severity. Hence, patients require varying levels of support and contact with healthcare professionals [25]. Findings from the previous feasibility study have indicated that a pre-defined number of sessions with the pharmacist may not be optimal for this group of patients [16]. Hence, the S-MAP intervention will be delivered in up to four sessions over a 4-month period with the number of sessions tailored to each patient’s needs. A flow chart detailing the session flow of the intervention is provided in Fig. 1.

Prior to session 1, the pharmacist will obtain a list of medications prescribed to the patient using the PMR and contact the patient’s general practitioner (GP) for clarification, if required. At session 1, the pharmacist will confirm the medication list with the patient and undertake an adherence assessment, using the app. This assessment will explore seven areas: (1) medication knowledge, (2) routine/organisational barriers, (3) practical barriers, (4) level of social support, (5) forgetfulness, (6) level of motivation and (7) cases of intentional non-adherence. This assessment tool was designed based on the previous TDF-based qualitative work [10] and modified based on feedback from participants in the previous feasibility study [16]. Identified adherence problems will be automatically mapped to recommended adherence solutions (i.e. BCTs) and, using a patient-centred (i.e. collaborative) approach, the pharmacist and patient will discuss which solution(s) would be most suitable. As part of the intervention, all patients will be offered a medication diary (BCT ‘self-monitoring’) which will contain details of their prescribed medications. A review session will be scheduled for 1–2 weeks later.

At session 2, the pharmacist will review the patient’s medication diary (if used), provide feedback on this (BCT: feedback on behaviour) and then re-assess adherence using the self-report measures used previously for screening. If adherence has improved, praise will be given by the pharmacist (BCT: social reward). No other BCTs will be delivered or recommended (unless additional support is requested) and the pharmacist will agree to contact the patient by telephone in 6–8 weeks for another review. If the patient is non-adherent at session 2, then the pharmacist will deliver or recommend additional BCTs and schedule a review session in the pharmacy in 6–8 weeks.

Session 3 may be conducted via telephone or in the pharmacy, depending on the patient’s adherence and need for more support at session 2. The pharmacist will review the patient’s diary if used (pharmacy sessions only) and re-assess adherence. If the patient is deemed adherent, then praise will be given and no other BCTs will be delivered. Non-adherent patients will be offered more support and then invited back for another review session in 6–8 weeks. Patients who are telephoned for session 3 and deemed non-adherent will be invited to attend the pharmacy as soon as possible and offered more support (and further review in 6–8 weeks).

Session 4 will only be offered to those patients who were deemed non-adherent (or required more support) at session 3. At the final intervention session (session 3 or 4 depending on adherence), patients will be advised to maintain contact with the pharmacy should they require ongoing support.

The app will guide each of the sessions and act as an intervention summary by allowing data on the sessions between patients and pharmacists (e.g. solutions recommended) to be captured. It is anticipated that sessions will last between 20 and 30 min, although this study will help to confirm this. GP practices will be informed about the study and may be contacted following the sessions if the issues cannot be resolved by the pharmacist (e.g. where a change to the prescription is recommended to improve adherence).

### Training package overview

A brief training package was delivered as part of the previous feasibility study [16] in a face-to-face session (1.5 h) at each pharmacy and found to be useful. However, additional training needs were identified (e.g. the need to practise elements of intervention delivery). To further explore the training needs of community pharmacists, in the context of providing adherence support to older adults, interviews (*n* = 15) and a survey of community pharmacists (*n* = 143) were conducted in NI as part of a linked study [16]. This research found that pharmacists required additional training to equip them with the skills required to deliver key components of the S-MAP intervention (e.g. goal-setting BCTs). In view of these findings, a modified training package will be developed and delivered in a more interactive format for this pilot study. This will consist of a 1-day workshop held in each of the two locations (a ‘distance learning’ package will be developed for pharmacists who are unable to attend). The training workshop will include didactic sessions that provide an overview of the intervention and study procedures as well as interactive sessions and activities (e.g. video demonstrations, role plays) (further information about the training workshop is available from the authors upon request). The training workshops will be audio-recorded (with consent) and pharmacists will have the opportunity to provide feedback on the training via a brief survey. A secure online discussion forum will be set up (on the distance learning platform Moodle) to allow study pharmacists to communicate directly with each other following the training and provide ongoing support for the study duration. Participating pharmacy support staff will be required to undertake training in study procedures via a ‘distance learning’ package and site visits by a researcher.

### Outcome data collection

Outcome data will be collected at baseline (following patient recruitment and pre-session 1) and at 6 and 12 months follow-up from baseline. The primary outcome of interest is medication adherence, which the literature recommends should be measured using multiple measures, one of which should be objective [26]. Therefore, adherence will be objectively measured using dispensing data from each pharmacy’s PMR to calculate each patient’s Medication Possession Ratio (MPR) and Daily Polypharmacy Possession Ratio (DPPR) [27, 28] in the 12 months pre- and post-session 1. Both measures attempt to examine the amount of medication that the patient has available over the defined period (as a surrogate for consumption). The two adherence questionnaires (MARS-5 [22] and one item adapted from Lu et al. [23]) used for screening will be used as a baseline measure and will be administered again at 6 and 12 months (via postal questionnaire). The adapted question from Lu et al. asks patients to pick one of six options ranging from very poor to excellent in response to the following statement: ‘Many people are not able to take all of their medicines as prescribed by their doctor. Rate your ability to take all of your regular prescribed medicines in the last month’ [23].

In addition to measuring adherence, the most recent Cochrane review on adherence interventions by Nieuwlaat et al. [3] recommends the inclusion of appropriate clinical and humanistic outcomes that are important to patients. Therefore, health-related quality of life (HRQoL) and unplanned hospitalisations, both of which have been selected to be part of a core outcome set for research conducted with older people prescribed several medications [29], will be measured in this study. The EQ-5D-5 L questionnaire (UK version) will be administered as a self-report measure of HRQoL [30]. Unplanned hospitalisations, resulting in an overnight stay in a hospital, will be measured via self-report from patients using a tool developed specifically for this study (which can be obtained from the authors upon request). This pilot study will also be used to determine whether it is feasible to cross-check this self-reported information on hospitalisations based on GP records. The questionnaires (measuring HRQoL and hospitalisations) will be administered at baseline by pharmacy staff (pre-session 1) and again at 6 and 12 months’ follow-up via postal questionnaire (along with the adherence measures). Patients will be telephoned as a reminder and given the opportunity to complete the questionnaires via telephone if necessary.

Analysis in relation to the study outcome measures will be descriptive as the study is not seeking to assess intervention effectiveness. Rather, the purpose is to test the feasibility of the data collection procedures in advance of a cRCT. Descriptive statistics (e.g. mean, standard deviation) will be conducted using SPSS (version 25.0).

### Process evaluation and progression criteria

A process evaluation will be embedded in the study to assess: (1) training fidelity and acceptability, (2) intervention fidelity and acceptability from the viewpoint of patients and pharmacists and (3) the mechanism of action of the intervention. Firstly, it will explore if the training is delivered by the researchers and received by the pharmacists as intended (training fidelity) and evaluate how acceptable this is to pharmacists. This will be assessed by audio-recording training workshops and a post-workshop feedback survey. Secondly, it will explore if the intervention was delivered by pharmacists (fidelity of intervention delivery) and received by patients as intended (fidelity of intervention receipt), by audio-recording, transcribing and coding all sessions held in the pharmacy for one patient (with their consent) per pharmacy. At the end of intervention delivery to patients, semi-structured qualitative interviews will be conducted with the recruited pharmacists to review acceptability of the intervention. Pharmacists will also be asked about their views on the training and support provided by the research team and study procedures. Interviews will be completed face-to-face at a convenient location (e.g. pharmacy site) or via telephone, audio-recorded (with consent) and transcribed verbatim. Patients will also be given a short post-intervention survey to complete to explore their views on the intervention including the support received from pharmacists, any materials provided during sessions, perceived benefits (or lack thereof) and their overall experience. The feedback from pharmacists and patients will inform future refinements to the intervention and study procedures in advance of a larger trial. Finally, the process evaluation will seek to identify how the intervention might work to bring about change in adherence behaviours (i.e. the mechanism of action) using qualitative and quantitative data collected from pharmacists and patients including audio-recordings of patient sessions, feedback interviews and surveys. Audio-recordings of patient sessions and post-intervention delivery feedback interviews with pharmacists will be coded to explore which BCTs were delivered by pharmacists and received by patients and which were most helpful in improving adherence. We will also explore, from session audio-recordings, any patient-reported changes in barriers to adherence (e.g. changes to medication knowledge, routine or side effects). As part of the post-intervention feedback survey, patients will be asked if they received a range of resources (e.g. the medication diary) and the usefulness of these resources on a scale of 1–5. This quantitative data (from patient surveys) and qualitative data (from patient session and pharmacist interview audio-recordings) will provide an indication as to the potential mechanism(s) of action of the intervention.

This pilot study will provide an indication as to whether a definitive cRCT of the S-MAP intervention is warranted and whether further modifications are required. The use of ‘Stop, Amend, Go’ progression criteria (also known as continuation criteria) for pilot studies has recently been advocated by Avery et al. [31]. The progression criteria that will be used in this study for decision-making purposes are presented in Table 3.

**Table 3:** Progression criteria (‘Stop’, ‘Amend’, ‘Go’) for the S-MAP pilot study

Concept	Data source(s)	Progression criteria		
		Stop (unless there are clear and modifiable contextual or design issues that account for this^a^)	Amend	Go
Pharmacy recruitment	Recruitment records held by the research team	If ≤ 5 pharmacies are recruited within 8 months	If 6–9 pharmacies are recruited and/or it takes longer than predicted (> 4–6 months)	If ≥ 10 pharmacies are recruited to take part in ≤ 4 months
Pharmacy retention	Retention records held by the research team	If ≤ 49% of pharmacies are retained for the required period	If 50–79% of pharmacies are retained for the required period	If ≥ 80% of pharmacies are retained for the required period
Patient recruitment	Study documentation completed by pharmacy staff	If ≤ 59 patients are recruited within 6 months^b^ or alternatively^c^ if ≤ 49% of pharmacies achieve a monthly recruitment rate of two patients per month for any three consecutive months	If 60–95 patients are recruited within 6 months^b^ or alternatively^c^ if 50–79% of pharmacies achieve a monthly recruitment rate of two patients per month for any three consecutive months	If ≥ 96 patients are recruited within 6 months^b^ or alternatively^c^ if ≥ 80% of pharmacies achieve a monthly recruitment rate of two patients per month for any three consecutive months
Patient retention	Study documentation completed by pharmacy staff	If ≤ 49% of patients are retained for the required period	If 50–79% of patients are retained for the required period	If ≥ 80% of patients are retained for the required period
Fidelity of pharmacist training package: delivery	Audio-recordings of pharmacist workshops	If ≤ 49% of planned training components are delivered by the researchers	If 50–79% of planned training components are delivered by the researchers	If ≥ 80% of planned training components are delivered by the researchers
Fidelity of pharmacist training package: receipt	Audio-recordings of pharmacist workshops	If ≤ 49% of delivered training components are received by pharmacists as intended	If 50–79% of delivered training components are received by pharmacists as intended	If ≥ 80% of delivered training components are received by pharmacists as intended
	Post-workshop feedback survey	If ≤ 49% of pharmacists report that they feel prepared to take part in the study	If 50–79% of pharmacists report that they feel prepared to take part in the study	If ≥ 80% of pharmacists report that they feel prepared to take part in the study
Acceptability of pharmacist training day	Post-workshop feedback survey	If ≤ 49% of pharmacists report that the training day was acceptable	If 50–79% of pharmacists report that the training day was acceptable	If ≥ 80% pharmacists report that the training day was acceptable
Fidelity of intervention delivery	Audio-recordings of a sample of patient sessions	If ≤ 49% of BCTs are delivered to patients when appropriate	If 50–79% of BCTs are delivered to patients when appropriate	If ≥ 80% of BCTs are delivered to patients when appropriate
Fidelity of intervention receipt	Audio-recordings of a sample of patient sessions	If ≤ 49% of delivered BCTs are received by patients as intended	If 50–79% of delivered BCTs are received by patients as intended	If ≥ 80% of delivered BCTs are received by patients as intended
Acceptability of intervention to pharmacists	Post-intervention delivery qualitative interviews	If ≤ 49% of pharmacists report that the intervention was acceptable	If 50–79% of pharmacists report that the intervention was acceptable	If ≥ 80% pharmacists report that the intervention was acceptable
Acceptability of intervention to patients	Post-intervention delivery feedback survey	If ≤ 49% of patients report that the intervention is acceptable	If 50–79% of patients report that the intervention is acceptable	If ≥ 80% of patients report that the intervention is acceptable
Enactment of treatment principles	Audio-recordings of a sample of patient sessions	If ≤ 49% of patients engaged with (or used) the delivered (or recommended) BCTs	If 50–79% of patients engaged with (or used) the delivered (or recommended) BCTs	If ≥ 80% of patients engaged with (or used) the delivered (or recommended) BCTs
Missing data	Data collected during the study (questionnaires, dispensing data)	If ≥ 50% of the main outcome data are missing	If 21–49% of the main outcome data are missing	If ≤ 20% of the main outcome data are missing

^a^This includes aspects of the study/intervention that may be modified in advance of a larger definitive trial

^b^To enable sufficient time to assess patient recruitment procedures, the patient recruitment period may be extended up to a maximum of 12 months (post-training) if major ethics amendments are made during the pilot study

^c^The alternative ‘rate-related’ criterion recognises that successful patient recruitment procedures may take some time to establish

The study will not proceed to a full-scale cRCT if one or more of the concepts in Table 3 meet the ‘Stop’ criterion unless there are clear and potentially modifiable contextual issues that account for the findings and/or the study procedures/intervention design could be amended to overcome any issues. Further feasibility and pilot testing may be deemed appropriate as an alternative to advancing directly to a cRCT if issues are identified, but it is unclear whether these are modifiable and if other aspects of the study and intervention show promise. The trial will proceed to a cRCT, with caution, if there are issues that can be remedied (i.e. one or more of the concepts in Table 3 meet the ‘Amend’ criteria). If there are no issues for concern that could potentially threaten the future success of the trial (i.e. all concepts in Table 3 meet the ‘Go’ criterion), then a future cRCT will be planned. If there is insufficient evidence available from the data to support a judgement for any of the concepts listed, then the ‘Stop, Amend, Go’ criteria will not be applied. For example, if it is unclear from the session audio-recordings whether patients engaged with or used the delivered/recommended BCTs, then the ‘enactment of treatment principles’ concept will not contribute to the final decision on whether to proceed to a cRCT.

### Data management and monitoring

All participants will receive a unique study ID number and data will be anonymised/pseudonymised (e.g. questionnaires and interview transcripts) as appropriate. No directly identifiable patient information (e.g. names, dates of birth) will be recorded on the app in order to protect patient confidentiality. Lockable storage boxes will be provided to each pharmacy site for securing study paperwork. Following intervention delivery, the researcher will collect study documentation from pharmacies, after which the research team will assume responsibility for its safekeeping. All hardcopy data (e.g. consent forms) will be stored in locked fire-resistant storage cabinets and electronic data securely stored on encrypted and password-protected computers and servers. At the end of the study, data will be retained for 5 years before being destroyed. Any data published as a result of this study will not be attributable to individual patients, pharmacy staff or their affiliated community pharmacy/company. Only a subset of the research team, who are responsible for data analysis, will have direct access to confidential data collected.

A Project Advisory Group (PAG), consisting of two patients and two pharmacy representatives, will assist the research team during the course of this pilot study by advising and commenting on the study design and progress. Any amendments to the protocol will be communicated to relevant parties (e.g. pharmacy sites, Research Ethics Committee, trial registries) and reported in the final published manuscript. The findings of this study will be communicated to all participants, published in relevant journals and presented at conferences.

## Discussion

The aim of this pilot study is to test the feasibility of the S-MAP intervention and study procedures in community pharmacies in NI and London. This will help to establish whether to proceed to a cRCT to assess intervention effectiveness. The intervention aims to improve older patients’ adherence to multiple medications through a tailored intervention that will be guided by a web-app which will be accessed via iPads. This study will also assess the feasibility of measuring outcomes (unplanned hospitalisations and HRQoL) that were included in a core outcome set for research conducted with older adults prescribed multiple medications in primary care [29].

Community pharmacists will be trained to deliver the intervention at a 1-day interactive workshop. This study will explore how acceptable this is to pharmacists and whether this equips them with the skills required to deliver the intervention. Findings from a recent meta-analysis conducted by Conn and Ruppar [11] have supported the decision to select community pharmacists as the intervention provider as adherence interventions delivered by pharmacists were shown to be more effective than those delivered by other healthcare professionals (nurses, clinicians). As referred to previously, this meta-analysis also highlighted that tailoring of adherence interventions on an individual basis is uncommon. Thus, this community pharmacy-based study will add to the literature by testing how the use of an app can guide this tailoring process which involves mapping adherence problems to the solutions most likely to bring about patient behaviour change. This systematic approach aims to ensure consistency in the delivery of intervention components and ultimately facilitate intervention replication in other clinical settings.

Previous research into adherence interventions has seldom utilised theory to better understand the underlying reasons for the success and failure of interventions [7]. We believe this is the first study to adopt a theory-based approach, using the Theoretical Domains Framework, to develop a tailored app-guided intervention to improve older patients’ adherence to multiple medicines. The embedded process evaluation will explore potential mechanisms of action of the intervention and provide insight into which components are likely to be effective or ineffective and the potential reasons why.

### Study status

This study was registered at ISRCTN (10.1186/ISRCTN73831533) on 12 January 2018. At the time of submission of this study protocol (version 5.0; date, December 7, 2018), the app development phase was complete and staff from 12 community pharmacies (15 pharmacists, 7 support staff) had been recruited and trained. Thirty-eight patients had also been recruited and intervention delivery commenced.

### Study amendments

A key objective of this study is to test approaches to the recruitment and retention of patients. The patient screening strategy has therefore been amended during the patient recruitment phase of the study (ongoing at the time of submission) based on feedback from participating pharmacists and the PAG. Pharmacists were reporting that the self-report adherence measure used for screening may not be an accurate reflection of their own assessments of patients’ adherence behaviour. This is supported by research showing that subjective self-report measures often overestimate adherence, in comparison with more objective measures (e.g. dispensing records) [32]. Therefore, the self-report measure has been removed from the eligibility screening process and instead, pharmacists will identify non-adherent patients, using pharmacy dispensing records or based on discussions with them. The adherence measures will now be administered along with the EQ-5D-5L and hospitalisations questionnaires after recruitment into the study (i.e. pre-session 1).

Some pharmacist participants have also reported that they feel patients aged 50 years and over, with long-term conditions, could also benefit from participation in this research. In addition, ethnic minority groups may be at a particular disadvantage from the age limit of 65 years as they are commonly diagnosed with conditions, such as cardiovascular conditions and diabetes, much earlier [33, 34]. Early intervention and developing positive adherence behaviours when patients are newly diagnosed may have health benefits that last into old age and consequently benefit an ageing population. Therefore, the age limit for participation has been lowered to 50 years. This is in line with the work of UK-based ageing charities (AgeNI, AgeUK) which support patients aged ≥ 50 years [35, 36]. These amendments have received ethical approval.

To allow sufficient time to assess new study procedures, the patient recruitment period was extended until 31 July 2019. As a result, the 12-month follow-up time point has been removed to ensure the study can be completed on time with the funding provided. Data will still be collected at 6 months post-baseline as planned.

## Additional file

**Additional file 1.** SPIRIT 2013 Checklist: Recommended items to address in a clinical trial protocol and related documents.

## Data Availability

Available upon request from the corresponding author.

## Abbreviations

BCT: Behaviour change technique
cRCT: Cluster randomised controlled trial
PMR: Patient medication record
S-MAP: Solutions for Medications Adherence Problems intervention
TDF: Theoretical Domains Framework
